# Risk of cardiovascular disease in Germany: results from GEDA 2022

**DOI:** 10.25646/13126

**Published:** 2025-05-21

**Authors:** Roma Thamm, Yong Du, Laura Neuperdt, Catarina Schiborn, Birga Maier, Anne Starker, Hannelore Neuhauser, Matthias B. Schulze, Christin Heidemann

**Affiliations:** 1 Robert Koch Institute Berlin, Department of Epidemiology and Health Monitoring; 2 German Institute of Human Nutrition Potsdam-Rehbrücke (DIfE), Department of Molecular Epidemiology; 3 German Centre for Diabetes Research (DZD); 4 German Centre for Cardiovascular Research (DZHK), Berlin site; 5 University of Potsdam, Institute of Nutritional Science

**Keywords:** Adults, Cardiovascular diseases, Heart attack, Risk factors, Stroke, Health status

## Abstract

**Background:**

Knowledge of the risk of cardiovascular disease (CVD) is important for its prevention.

**Methods:**

Data from a non-clinical test for the absolute risk of having a heart attack or stroke for the first time in the next ten years is available from 3,271 35- to 69-year-old participants in the GEDA 2022 study without a diagnosis of heart attack or stroke. This risk was categorised as *low* (< 5 %), *still low* (≥ 5 % – < 7.5 %), *increased* (≥ 7.5 % – < 10 %) and *high* (≥ 10 %). In addition, the self-perceived CVD risk was asked as *almost no, low, moderate* and *high risk.*

**Results:**

According to the CVD test, 73.5 % of adults were at low risk, 7.8 % were still at low risk, 6.0 % were at increased risk and 12.8 % were at high risk. In contrast, 28.7 % perceived themselves to be at almost no risk, 45.3 % at low risk, 20.4 % at moderate risk and 5.6 % at high risk of CVD. The higher the test-based risk, the lower the proportion of those who perceived themselves as having almost no or only a low risk. Nevertheless, half of the people with an increased to high risk according to the test result perceived themselves to be at almost no or only a low risk. The underestimation of risk was associated with lower education, better mental health and physical activity in both sexes.

**Conclusions:**

People who underestimate their risk of CVD despite an unfavourable risk factor profile are a key target group for cardiovascular prevention.

## 1. Introduction

Cardiovascular diseases (CVD) are the main causes of morbidity and mortality in Germany and are associated with correspondingly high medical costs [1, 2]. The most important cardiovascular events include heart attacks and strokes, which are often caused by arteriosclerotic plaque deposits in the arterial walls and the resulting circulatory disorders. The reduction of risk factors is considered an important preventive measure for the development of CVD [3]. Significant modifiable risk factors for CVD are cardiometabolic diseases such as high blood pressure, diabetes mellitus, lipid metabolism disorders and obesity, as well as health-impairing behaviour such as smoking, an unhealthy diet or physical inactivity. The main risk factors that cannot be modified are age, sex and genetic factors. Overall, this results in a risk of CVD that plays a significant role in the planning and implementation of preventive measures from an individual, medical and health policy perspective. The risk of CVD and its distribution according to age, sex, social and regional situation are therefore of particular interest for health monitoring and health reporting in Germany.

While initially only the number of risk factors was counted, algorithms were developed over thirty years ago to better assess the risk of CVD [4], which have also found their way into medical guidelines for cardiovascular prevention and treatment. The current guidelines on cardiovascular prevention of the German Society of Cardiology and the German Society of General Medicine provide differentiated recommendations for the medical determination of the overall cardiovascular risk in people without a previous cardiovascular event from middle age [5, 6]. In Germany, various risk scores are used for this purpose, in particular the SCORE2 (in an earlier version SCORE-Germany) [7], the PROCAM score [8] and the arriba score [9]. A number of other scores are used internationally [10–12]. What all these scores have in common is that clinical values, i.e. usually laboratory parameters measured by a doctor, primarily cholesterol and blood pressure values, are necessary for risk prediction. As a result, these methods are not well suited or easily accessible to laypersons due to the use of clinical parameters. Surveillance of the risk of CVD at population level is also made more difficult by the need for examination surveys with laboratory tests and measurements, which are significantly more complex and therefore less often feasible compared to questionnaire surveys.

Therefore, non-clinical risk scores based on information on risk factors that are easy to record are of interest. This is the case with the cardiovascular disease risk test recently developed for Germany by the German Institute of Human Nutrition Potsdam-Rehbrücke (DIfE), which is based solely on questions about age, sex and lifestyle-associated factors, the presence of hypertension and diabetes, and family history of CVD. In a validation study in which the prediction of the CVD risk test was compared with the actual occurrence of cardiovascular events, the risk test showed a similar predictive quality to established clinical prediction models [13, 14].

Various psychological models of health behaviour regard the perception of risks as one of the key parameters with regard to possible changes in behaviour. It is based on analytical, emotional and experience-based assessments that influence health behaviour. A central assumption is that people with a higher perception of risk are more likely to change their behaviour. Static models such as the *Health Belief Model* or the *Theory of Planned Behaviour* are based on the assumption that the perception of health risks influences the motivation to change behaviour. These models primarily consider the motivational phase that leads to an intention. Dynamic models such as the *Transtheoretical Model* also consider the volitional phase, which involves planning, initiating action and maintaining the new behaviour. These models are crucial for the development of behaviour change interventions [15–17]. In view of the fact that perceived risks can differ from actual risks, it is relevant to analyse the determinants of the discrepancy. The subject of this article is therefore the determination of the 10-year risk of a heart attack or stroke using the CVD risk test developed by the DIfE in a nationwide sample of the 35- to 69-year-old general population without a previous heart attack or stroke and the comparison with the risk assessed by the respondents themselves. In addition, the article analyses the characteristics of people who underestimate their CVD risk.

**Key messages**

► The risk of having a heart attack or stroke for the first time in the next ten years can be measured population-wide using a validated, non-clinical risk test based on survey data.
► According to the risk test, 6.0 % of 35- to 69-year-olds had an increased risk of cardiovascular disease and 12.8 % had a high risk.
► 20.4 % of adults rated their risk of having a heart attack or stroke for the first time in the next ten years as moderate, 5.6 % rated it as high.
► Half of the people with an increased to high risk according to the test result had almost no or only a low risk in their own perception.
► This underestimation of one’s own risk of illness was associated with lower education, better mental health and physical activity in both sexes.

## 2. Method

### 2.1 Study design and sample

The study German Health Update (GEDA) was conducted on behalf of the Federal Ministry of Health as a nationally representative cross-sectional study as part of the RKI health monitoring programme [18, 19]. The German-speaking adult resident population in Germany was surveyed. The data collection for GEDA 2022 was carried out as a modular, standardised computer-assisted telephone interview by landline or mobile phone from February 2022 to January 2023 with adults aged 18 and over [20]. Data from one of the four topic-specific modules, which was conducted from June 2022 to January 2023 with 5,796 participants, was used for the analyses on the risk of CVD. Participants who stated that they had already been diagnosed with a heart attack or stroke during their lifetime (*n* = 416) or did not answer the question (*n* = 7) were excluded from the analyses. As the CVD risk test is validated for people aged 35 and over to under 70 [14], people aged 18 to 34 (*n* = 708) and people aged 70 and over (*n* = 1,394) were also excluded from the analyses. Thus, data from 3,271 participants aged 35 to 69 years without a known heart attack or stroke were analysed for this article.

### 2.2 Test-based and self-perceived 10-year CVD risk

To determine the test-based CVD risk, the DIfE’s CVD risk test was used, which estimates the probability of the first occurrence of a heart attack or stroke in the next ten years. The risk test calculates the absolute risk, i.e. the probability of the event occurring in an individual person within the defined period (with values between 0 % and 100 %). The simplified, non-clinical version of the questionnaire was used [13], the performance of which was analysed in EPIC Potsdam and EPIC Heidelberg and was comparable to the more detailed, non-clinical version. The following response categories were considered for the components of the simplified questionnaire version:

► Age (< 35, 35 – 39, 40 – 44, 45 – 49, 50 – 54, 55 – 59, 60 – 64, 65 – 69, 70 – 74, ≥ 75 years)
► Sex (female, male)
► Waist circumference (< 75, 75 – 79, 80 – 84, 85 – 89, 90 – 94, 95 – 99, 100 – 104, 105 – 109, 110 – 114, 115 – 119, ≥ 120 cm)
► Smoking (currently < 20 or ≥ 20 cigarettes/day, formerly < 20 or ≥ 20 cigarettes/day, never)
► Hypertension diagnosis (yes, no)
► Diabetes diagnosis (yes, no)
► Cardiovascular disease in biological parents (no or not known, yes – one parent, yes – both parents)
► Cardiovascular disease in at least one biological sibling (no or not known, yes)
► Consumption of wholemeal bread/muesli (0, 1, 2, 3, 4, >4 slices or portions/day)
► Consumption of red meat (never or rarely, 1 – 2 times/week, 3 – 4 times/week, 5 – 6 times/week, daily, several times daily)
► Coffee consumption (0 – 1, 2 – 5, > 5 cups/day)
► Consumption of sugar-sweetened beverages (never or not daily, 1 – 2, 3, 4, > 4 glasses/day)
► Consumption of vegetable oil (0 – 0.5, >0.5 – 1, > 1 – 2, > 2 tablespoons/day)

Waist circumference was calculated from information on height and weight, sex and age [21]. The response categories of the individual components are associated with a certain number of points, which increases with male sex, higher age, larger waist circumference, more intensive smoking, the presence of high blood pressure, diabetes and a family history of CVD, as well as higher consumption of red meat and sugar-sweetened beverages, but decreases with higher consumption of wholemeal products, coffee and vegetable oil [22]. The sum of all points for each person in the study population was first translated into the percentage absolute risk of CVD according to the following formula:



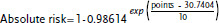



The calculated risk was then categorised as *low* (< 5 %), *still low* (≥ 5 % to < 7.5 %), *increased* (≥ 7.5 % to < 10 %) and *high* (≥ 10 %) [14, 23]. In line with the question on the self-perceived risk of diabetes, which was based on an internationally established formulation [21, 24], the self-perceived risk of CVD was recorded using the following question: ‘How would you rate your risk of having a heart attack or stroke in the next 10 years?’ with the answer options: *‘almost no risk’, ‘low risk’, ‘moderate risk’, ‘high risk’.*

### 2.3 Demographic, social and health-related characteristics

In addition to information on age and biological sex, information on education, categorised as low, medium and high education (defined by the Comparative Analysis of Social Mobility in Industrial Nations (CASMIN) classification system [25]), was taken into account. In addition, information on the region of residence in Germany, categorised as north-west, centre-west, north-east, centre-east and south [26], as well as on the settlement structure type, grouped as an independent city, urban district, rural district and sparsely populated rural district, was prepared for the analyses. The characteristic of living alone (yes, no) was derived from the answer to the question of how many people live in the household. The extent of social support (low: 3 – 8 points, medium: 9 – 11 points, strong: 12 – 14 points) was determined using the Oslo Scale (Oslo-3 Social Support Scale, OSS-3 [27]). The latter two characteristics were included as living alone and a lack of social support can increase the risk of cardiovascular disease [28, 29]. A high level of social support can be associated with positive effects on the cardiovascular system [29–31] and improve overall cardiovascular well-being [32] through stress reduction, lowering blood pressure and promoting health-related behaviours, among other things. The question: ‘How is your health in general?’ was used to measure self-rated general health [33]. The five response categories were dichotomised into good/very good and fair/poor/very poor. Self-rated mental health was recorded with the question: ‘How would you describe your mental health in general?’ [34]. The five response categories were also dichotomised into very good/excellent and good/less good/poor. Both operationalisations were carried out according to national and international standards [35–38]. Furthermore, the study participants were asked about their physical activity by answering the question: ‘Are you physically active for at least 5 hours per week? This includes, for example, sport, gardening and cycling.’ with the answer options ‘yes’ or ‘no’.

### 2.4 Statistical analyses

The prevalence of low, still low, increased and high CVD risk estimated by the CVD risk test was determined overall and stratified by sociodemographic and health characteristics, each with 95 % confidence intervals (CI). Differences between groups were considered statistically significant at a *p*-value of < 0.05 of the corrected Rao-Scott chi-square test or non-overlapping 95 % CI. The prevalence of self-perceived CVD risk was also calculated in the categories of almost no risk, low, moderate and high risk overall and stratified by sex. A Poisson regression was used to characterise people who underestimated their CVD risk. These are people who have an increased to high risk according to the CVD risk test, but who perceive themselves as having almost no or only a low risk. The multivariable analysis provided prevalence ratios (PR), i.e. the probability of the presence of the respective determinants mentioned below in the group of people with risk underestimation compared to the corresponding probability in the group of people without risk underestimation (where a value of 1 indicates no difference, values < 1 indicate a lower probability and values >1 indicate a higher probability), with 95 % CI and *p*-value [39]. When modelling the risk underestimation, age and sex as well as education, self-rated general and mental health, social support, physical activity, living alone and region of residence were considered as potential determinants. Interactions of these potential determinants with sex were tested in order to analyse any sex-related differences. Based on the assumption that people with prevalent diabetes or high blood pressure, for example, are more likely to realistically assess their own CVD risk, all other components of the CVD risk test were also considered in an extended model.

The statistical analyses were carried out using the statistical software STATA (version 17). The GEDA study design was observed by using the survey procedures in STATA. To ensure that the results are representative at national level, a weighting factor was applied in all analyses that considers the probability of participants being drawn and corrects for the population structure of Germany with regard to sex, age, federal state and education. The data from the Federal Statistical Office as at 31 December 2020 was used. The education distribution was taken from the 2018 microcensus.

## 3. Results

Table 1 shows the general characteristics of the study population. The average age was 52.4 years. 50.9 % of the participants were female, 49.1 % male. The proportion of people with a low level of education was 23.9 %. 69.3 % reported good to very good general health and 40.4 % reported very good to excellent mental health. 12.9 % of people reported a low level of social support. Three quarters (74.5 %) of the people reported that they were physically active for at least five hours a week and around one third (32.3 %) lived alone.

The estimated risk of having a heart attack or stroke for the first time within the next ten years based on the CVD risk test was low in 73.5 % of 35- to 69-year-olds, still low in 7.8 %, increased in 6.0 % and high in 12.8 %. People with an increased or high risk of a heart attack or stroke were significantly more likely to be older than 50, three and four times more likely to be male (9.2 % and 20.5 % respectively) than female (2.8 % and 5.4 % respectively) and twice as likely to be under (9.9 % and 20.5 % respectively) as physically active for at least five hours a week (4.7 % and 10.3 % respectively).

In addition, people at high risk of CVD were significantly more likely to have low (23.3 %) than medium (10.8 %) or high education (6.6 %), moderate to very poor (22.1 %) than good to very good self-rated general health (8.8 %) and good to poor (15.5 %) than very good to excellent self-rated mental health (9.0 %). In addition, they were about twice as likely to report low (22.3 %) as moderate (13.2 %) or strong (10.2 %) social support and were more likely to live alone (21.5 % vs. 8.8 %). In terms of region of residence, there was only a difference between the centre-west (17.0 %) and south (9.0 %) regions (Table 2).

**Figure 1:**
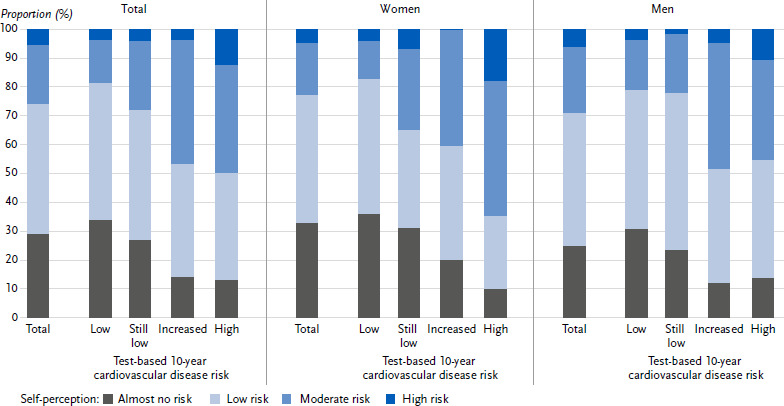
Perceived 10-year cardiovascular disease risk and proportions across categories of risk estimated by the CVD risk test

Figure 1 shows the prevalence of self-perceived CVD risk overall and in relation to the categories of risk estimated by the CVD risk test, both overall and by sex. 28.7 % of adults aged 35 to 69 stated that they perceived their own CVD risk as virtually non-existent. This proportion was higher for women than for men (32.6 % vs. 24.6 %). 45.3 % of adults perceived their CVD risk as low, 20.4 % as moderate and 5.6 % as high. The prevalence of a self-perceived low, moderate or high risk of CVD was about the same for women and men.

In the four groups formed according to test-based risk, there were different proportions for the categories of self-perceived risk. The proportion of those who perceived almost no or only a low CVD risk was 81.1 % in the group with a low risk according to the test. The higher the test-based risk, the lower this proportion became: in the still low-risk group it was 72.0 %, in the increased-risk group 53.1 % and in the high-risk group 50.1 %. The overall decrease across the four groups was significantly more pronounced in women than in men (-47.6 % vs.-24.4 %).

**Figure 2:**
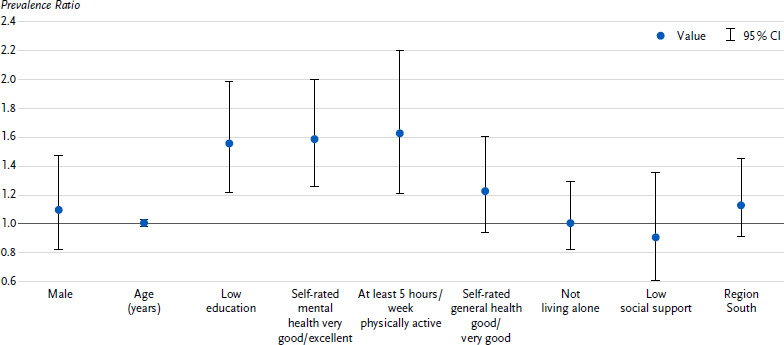
Characteristics of persons with an increased to high risk according to the CVD risk test, who perceived themselves to be at almost no to only a low risk of CVD; prevalence ratio with 95 % confidence interval (*n* = 152 women, *n* = 430 men)

Of the people with an increased to high risk according to the test result (18.8 % of all people), half (51.0 %) had almost no or only a low risk in their own perception. These people were included in the Poisson model to characterise people who underestimated their CVD risk. The probability of underestimating risk was higher for people with low education (PR 1.56; 95 % CI 1.22 – 1.99), for people with very good to excellent self-rated mental health (1.59; 1.26 – 2.00) and for people who reported being physically active for at least five hours per week (1.63; 1.21 – 2.20) (Figure 2). As self-rated general health was only a statistically significant determinant for men and living alone only for women (*p* < 0.05 for the interaction terms with sex), sex-related models were calculated. It was found that, in addition to the determinants mentioned, men with good to very good self-rated general health (1.48; 1.07 – 2.05) and women who did not live alone (1.80; 1.21 – 2.68) were more likely to underestimate their risk of CVD (Annex Figure 1a, Annex Figure 1b). The factors age, sex, social support and region of residence had no independent influence on the underestimation of one’s own CVD risk. The extended regression model, which also took into account all other components of the CVD risk test, revealed only previous smoking as a further determinant of risk underestimation (1.38; 1.04 – 1.83).

## 4. Discussion

In 2022, 6.0 % of 35- to 69-year-olds in Germany without a previous diagnosis of heart attack or stroke had an increased and 12.8 % a high absolute risk of having a heart attack or stroke in the next ten years, based on the risk estimate using the validated DIfE-CVD risk test [13, 14]. People of male sex, aged 50 and over or with a low level of education, people with a moderate to very poor self-rated general health or a good to poor self-rated mental health, people with less than five hours of physical activity per week and people with low social support, people living alone and people from the centre-west region were comparatively more likely to have a high risk of CVD. These results are particularly relevant for the derivation of sex-related measures in the area of risk communication. According to a meta-analysis by Bakhit et al., communicating information on cardiovascular risk reduced overall risk factors and increased self-perception. The communication of cardiovascular risk should be an integral part of routine consultations [40].

The distribution of the risk of CVD in the German population based on a risk score has only been analysed once so far. The clinical SCORE risk score, which is no longer in use, and examination data from the nationwide DEGS1 study were used. The analysis of adults aged 40 to 69 years revealed a proportion of 13.4 % with a high cardiovascular risk; however, the endpoint was the 10-year risk of cardiovascular mortality [41]. The CVD risk test, on the other hand, can be used in nationwide survey studies without the need for costly and time-consuming collection of clinical parameters. The surveillance of non-communicable diseases (NCD surveillance) in the health reporting web portal (https://gbe.rki.de/) already shows the average absolute 10-year CVD risk for the German adult population aged 18 and over in 2022 [42]. The development of a time series on CVD risk in conjunction with time series of other key indicators on morbidity and mortality for CVD [43, 44] and numerous risk factors, including diabetes [45] is planned and will contribute to the assessment of cardiovascular health in Germany.

The actual risk often differs from the self-perceived risk. The understanding and interpretation of risk are not necessarily rational processes and depend heavily on the way in which risk is communicated and how the individual deals with the corresponding health threat [46, 47]. With regard to cardiovascular disease, risk misjudgement and frequent risk underestimation have already been identified in other studies [48–50], the extent of which also depends heavily on the operationalisation of risk estimates and the communication and presentation of risk tools [51]. In the present study, one fifth (20.4 %) of adults perceived their CVD risk as moderate and 5.6 % as high. The combination of test-based and self-perceived risk showed that the higher the risk, the lower the proportion of those who perceived themselves as having almost no or only a low risk. Nevertheless, half of the people with an increased to high risk according to the CVD risk test assessed their own risk as almost non-existent or only low. A similar order of magnitude was reported by Oertelt-Prigione et al. based on the Berlin Women’s Risk Evaluation (BEFRI) study. The study compared the subjective perception of cardiovascular risk of 1,066 women aged 25 to 74 years with their actual risk assessment according to a clinical Framingham score and showed that 49 % of women underestimated their cardiovascular risk [52]. In contrast, in a study conducted exclusively among smokers, the overestimation of risk by those affected predominated [53].

Possible reasons discussed for a misjudgement of cardiovascular risk are that people have a certain amount of knowledge about the causes of the diseases, but only partially use this knowledge to link individual personal risk factors with an increased risk of disease. In particular, there may be an assumption that actions that contribute to risk (e.g. smoking) are outweighed by risk avoidance measures (e.g. sufficient physical activity). However, knowledge of the risk factors and realistic ideas about one’s own CVD risk are not only prerequisites for positive primary preventive behavioural changes [15, 54, 55], but also play an important role in improved secondary preventive behaviour, in addition to the conviction that the disease risk can be influenced and awareness of the severity of the disease. For example, individuals with cardiovascular disease who correctly assessed their risk of recurrent events reported higher rates of smoking cessation and more frequent use of antihypertensive therapies and statins [56]. In our study, people with a low level of education, people who felt very well mentally and people who reported being physically active for at least five hours per week in particular underestimated their risk of CVD despite an unfavourable risk factor profile. The regression model extended to include the CVD test components also showed that existing diseases such as diabetes and high blood pressure were not associated with a misjudgement of one’s own CVD risk, just like the other components, with the exception of previous smoking, which was positively associated. The results of international studies on determinants of misperceptions of cardiovascular risk are heterogeneous, with physical activity and low income, for example, being repeatedly associated with a risk perception that was too low compared to the actual risk [57–60]. Studies of patients with CVD have also shown that those with higher education are more likely to be aware of their risk factors or measurements [61] and have better health literacy [62] than those with lower education. With regard to the positive association of very good to excellent self-rated mental health with underestimation of CVD risk, it is striking that good mental health is associated with better cardiovascular health [63], but awareness of CVD risk factors can also be less favourable. Overall, a so-called optimism bias may play a role in the underestimation of risk [64].

Simultaneous recording of test-based and self-perceived risk has already been implemented for Germany as part of the population-representative survey study Disease Knowledge and Information Needs – Diabetes mellitus (2017) [65] for the 5-year diabetes risk [21]. In addition to characterising people at high risk, a key finding was that the self-perceived diabetes risk was low, even if it was high according to the Diabetes Risk Test. The analysis emphasised the relevance of risk communication, particularly on the part of healthcare professionals for people at high risk of diabetes, as an important component for the success of prevention measures. The presented findings on test-based and self-perceived CVD risk also emphasise this need for CVD. As with diabetes, people at high risk of cardiovascular disease, who in their own perception have almost no or only a low risk, are an important target group for preventive measures. For these people in particular, for example, access to a low-threshold risk test, i.e. one that is based solely on requested information and that they can carry out themselves, can be of great benefit. This is because the test evaluation not only provides information about the personal risk, but also shows individual starting points for reducing the risk and thus preventing the development of the disease. The CVD risk self-test can be accessed online via the DIfE [22] as well as in several languages via the website of diabinfo, the information portal on diabetes in Germany [66]. The use of the risk score can also be advantageous for use in medical practice for individual risk assessment and risk communication and can be combined with targeted recommendations for behaviour-related prevention or the use of prevention courses [67]. In addition to the non-clinical test version, an extended clinical test version with systolic and diastolic blood pressure as well as total and HDL cholesterol as additional test components is also available for the medical context [14].

## Limitations

The GEDA 2022 study was conducted as a telephone survey. Therefore, misclassifications cannot be completely ruled out. For example, waist circumference, as a component of the CVD risk test, could not be measured, but was calculated on the basis of a regression equation including self-reported weight and height, in line with the procedure used in previous analyses [21]. Self-reported body weight and height can be distorted per se in that body weight is often underestimated compared to standardised measurements, while height tends to be overestimated [68]. In addition, information on smoking behaviour and physical activity could be distorted by socially desirable response behaviour, which may lead to an underestimation of current smoking or an overestimation of physical activity. The question on the recording of physical activity also deviates from the complex recording of compliance with the current physical activity recommendations of the World Health Organisation and the national recommendations for physical activity and the promotion of physical activity for adults [69]. However, the question is part of the established German Diabetes Risk Test [70]. In addition, the scales of predicted and self-perceived CVD risk differ. While the quantitative estimation using the CVD risk test resulted in absolute risks in per cent and these were then divided into four risk categories, the question about the self-assessment of the risk of having a heart attack or stroke for the first time in the next ten years led to a qualitative assessment with the four specified risk categories as answer options. In addition, a weighting factor was applied in all statistical analyses, which considers the probability of the participants being drawn and corrects for the population structure of Germany with regard to the characteristics of sex, age, federal state and education. Nevertheless, a selection bias due to non-response cannot be completely ruled out.

## Conclusion

Around a fifth of 35- to 69-year-old adults in Germany without a diagnosis of heart attack or stroke are at an increased to high risk of having a heart attack or stroke in the next ten years. Half of people with an unfavourable risk factor profile underestimate their own risk of a heart attack or stroke. This clearly shows the need for both individual and societal prevention to avoid CVD, already in middle age. These include, for example, targeted education about the negative effects of modifiable risk factors in the areas of diet, exercise, alcohol and tobacco consumption or the creation of framework conditions that reduce harmful behaviour, for example through legal regulations such as the strengthening of tobacco control measures, the expansion of cycle paths and footpaths for more exercise in everyday life and the reduction of taxes on health-promoting foods (https://www.gbe.rki.de/DE/Themen/Rahmenbedingungen/rahmenbedingungen_node.html). In addition, measures aimed at increasing the utilisation of regular preventive check-ups, in which risk testing is an integral part, or the integration of tests in corresponding nationwide campaigns could help to improve risk awareness.

## Figures and Tables

**Annex Figure 1a:**
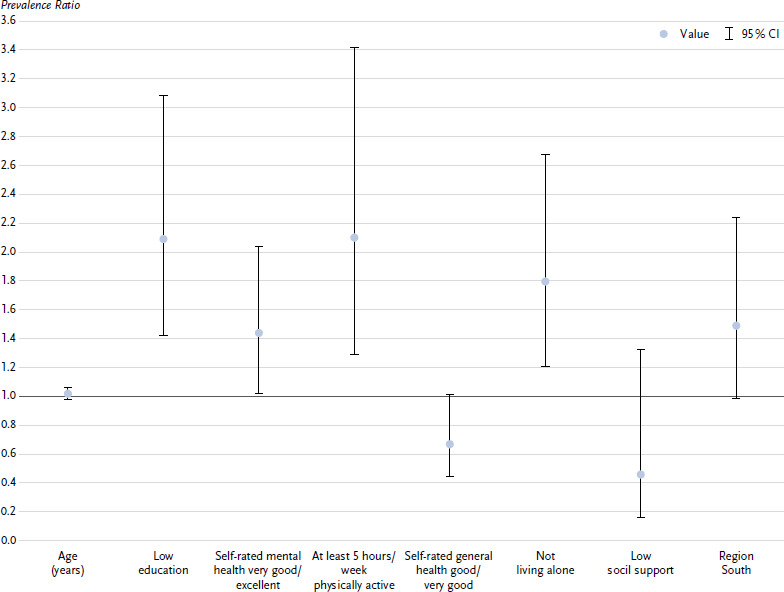
Characteristics of women with an increased to high risk according to the CVD risk test, who perceived themselves to be at almost no to only a low risk of CVD; prevalence ratio with 95 % confidence interval (*n* = 152)

**Annex Figure 1b:**
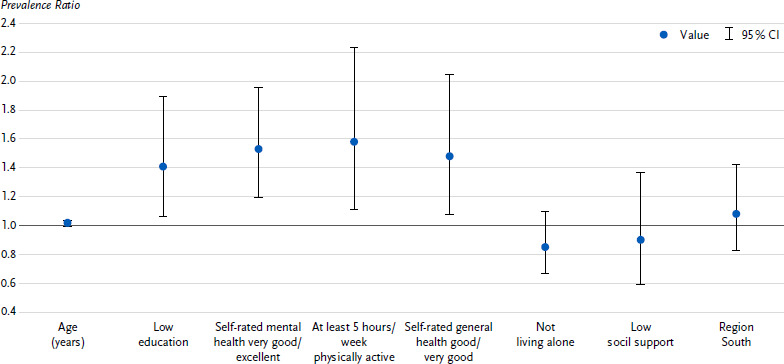
Characteristics of men with an increased to high risk according to the CVD risk test, who perceived themselves to be at almost no to only a low risk of CVD; prevalence ratio with 95 % confidence interval (*n* = 430)

**Table 1:** Characteristics of the 35- to 69-year old study population (*n* = 1,812 women, *n* = 1,459 men). Source: GEDA 2022

	*n*	%	(95 % CI)
**Sex**			
Female	1,812	50.9	(48.1 – 53.6)
Male	1,459	49.1	(46.4 – 51.9)
**Age group (years)**			
35 – < 50	900	40.9	(38.2 – 43.8)
50 – 69	2,371	59.1	(56.2 – 61.8)
Age (years)*	3,271	52.4	(51.9 – 53.0)
**Education**			
Low	403	23.9	(21.3 – 26.8)
Medium	1,510	55.1	(52.4 – 57.8)
High	1,347	21.0	(19.4 – 22.7)
**Self-rated general health**			
Good/very good	2,395	69.3	(66.6 – 71.9)
Fair/poor/very poor	873	30.7	(28.1 – 33.4)
**Self-rated mental health**			
Very good/excellent	1,489	40.4	(37.8 – 43.1)
Good/less good/poor	1,770	59.6	(56.9 – 62.2)
**Social support**			
Low	287	12.9	(10.9 – 15.2)
Medium	1,376	43.0	(40.3 – 45.8)
Strong	1,511	44.1	(41.4 – 46.9)
**At least 5 hours/week physically active**			
Yes	2,546	74.5	(71.9 – 76.9)
No	721	25.5	(23.1 – 28.1)
**Living alone**			
Yes	835	32.3	(29.6 – 35.1)
No	2,428	67.7	(64.9 – 70.4)
**Settlement structure type**			
Independent city	1,071	28.2	(25.7 – 30.7)
Urban district	1,177	39.1	(36.3 – 41.8)
Rural district	495	17.9	(15.9 – 20.2)
Sparsely populated rural district	399	14.9	(12.9 – 17.0)
**Region**			
North-west	539	16.2	(14.4 – 18.3)
Centre-west	1,093	34.7	(32.1 – 37.4)
North-east	347	9.6	(8.1 – 11.3)
Centre-east	330	10.6	(9.1 – 12.3)
South	962	28.8	(26.4 – 31.4)

CI = Confidence interval

*Mean

**Table 2:** Test-based 10-year cardiovascular disease risk in 35- to 69-year-olds, by sociodemographic and health-related characteristics (*n* = 1.812 women, *n* = 1.459 men). Source: GEDA 2022

	Low (< 5 %)			Still low (≥ 5 % to < 7.5 %)			Increased (≥ 7.5 % to < 10 %)			High (≥ 10 %)			*p*-value
	%	(95 % CI)	*n*	%	(95 % CI)	*n*	%	(95 % CI)	*n*	%	(95 % CI)	*n*	
**Total**	**73.5**	**(70.9 – 75.8)**	**2,152**	**7.8**	**(6.6 – 9.1)**	**315**	**6.0**	**(4.8 – 7.5)**	**204**	**12.8**	**(11.0 – 14.9)**	**378**	
**Sex**													< 0.001
Female	84.9	(82.1 – 87.3)	1,397	6.9	(5.3 – 8.8)	138	2.8	(2.0 – 4.0)	66	5.4	(3.9 – 7.5)	86	
Male	61.6	(57.4 – 65.5)	755	8.7	(7.0 – 10.7)	177	9.2	(7.0 – 12.0)	138	20.5	(17.3 – 24.1)	292	
**Age group**													< 0.001
35 – < 50 years	95.1	(91.6 – 97.2)	803	0.9	(0.3 – 2.4)	6	1.6	(0.6 – 4.4)	7	2.4	(1.1 – 5.4)	9	
50 – 69 years	59.0	(55.7 – 62.3)	1349	12.3	(10.5 – 14.4)	309	8.9	(7.1 – 11.0)	197	19.8	(17.1 – 22.7)	369	
**Education**													< 0.001
Low	57.3	(50.2 – 64.2)	192	10.6	(7.4 – 14.9)	52	8.8	(5.6 – 13.7)	38	23.3	(17.8 – 29.8)	93	
Medium	77.5	(74.3 – 80.3)	1,005	6.5	(5.1 – 8.2)	131	5.2	(3.8 – 7.1)	87	10.8	(8.8 – 13.3)	178	
High	81.0	(78.2 – 83.4)	949	8.1	(6.6 – 10.0)	132	4.3	(3.2 – 5.7)	78	6.6	(5.1 – 8.5)	106	
**Self-rated general health**													< 0.001
Good/very good	79.3	(76.6 – 81.8)	1,701	7.1	(5.9 – 8.7)	216	4.7	(3.6 – 6.2)	132	8.8	(7.0 – 11.0)	198	
Fair/poor/very poor	59.9	(54.4 – 65.1)	451	9.2	(6.8 – 12.3)	99	8.8	(6.1 – 12.6)	72	22.1	(179 – 26.9)	179	
**Self-rated mental health**													0.005
Very good/excellent	77.6	(74.1 – 80.8)	1,041	7.4	(5.8 – 9.5)	141	5.9	(4.1 – 8.4)	85	9.0	(6.9 – 11.6)	136	
Good/less good/poor	70.6	(67.0 – 73.9)	1,106	7.9	(6.4 – 9.8)	172	6.0	(4.5 – 8.0)	119	15.5	(12.8 – 18.5)	242	
**Social support**													0.019
Low	63.5	(53.9 – 72.2)	164	8.0	(4.7 – 13.4)	25	6.2	(3.0 – 12.3)	14	22.3	(15.0 – 31.7)	55	
Medium	73.2	(69.3 – 76.8)	890	7.7	(5.9 – 10.0)	143	5.9	(4.2 – 8.2)	100	13.2	(10.4 – 16.5)	161	
Strong	77.0	(73.5 – 80.1)	1,054	7.4	(5.8 – 9.3)	136	5.4	(3.8 – 7.7)	84	10.2	(8.0 – 12.9)	151	
**At least 5 hours/week physically active**													< 0.001
Yes	77.1	(74.5 – 79.6)	1,733	7.9	(6.6 – 9.5)	262	4.7	(3.6 – 6.1)	147	10.3	(8.5 – 12.4)	248	
No	62.4	(56.2 – 68.1)	417	7.2	(5.0 – 10.2)	53	9.9	(6.7 – 14.5)	57	20.5	(15.9 – 26.1)	129	
**Living alone**													< 0.001
Yes	61.8	(56.1 – 67.2)	475	8.6	(6.3 – 11.7)	79	8.0	(5.5 – 11.7)	66	21.5	(17.1 – 26.8)	154	
No	78.9	(76.4 – 81.1)	1,673	7.4	(6.1 – 8.9)	236	5.0	(3.8 – 6.6)	138	8.8	(7.3 – 10.5)	224	
**Settlement structure type**													0.689
Independent city	72.8	(67.6 – 77.5)	726	7.5	(5.3 – 10.7)	93	6.3	(4.0 – 9.9)	66	13.3	(9.8 – 17.7)	119	
Urban district	74.7	(70.6 – 78.4)	769	6.3	(4.9 – 8.1)	113	5.6	(4.0 – 7.7)	80	13.4	(10.4 – 17.1)	143	
Rural district	74.9	(68.9 – 80.0)	329	9.0	(6.2 – 13.0)	50	5.9	(3.7 – 9.1)	33	10.2	(6.7 – 15.2)	44	
Sparsely populated rural district	68.9	(61.6 – 75.4)	246	10.6	(7.2 – 15.3)	50	7.1	(3.5 – 13.9)	17	13.3	(9.5 – 18.4)	60	
**Region**													0.038
North-west	76.9	(71.3 – 81.8)	357	7.4	(5.0 – 10.8)	49	5.6	(3.0 – 10.4)	22	10.0	(7.2 – 13.8)	66	
Centre-west	70.5	(65.7 – 74.8)	702	7.7	(5.7 – 10.4)	104	4.8	(3.5 – 6.7)	77	17.0	(13.2 – 21.6)	149	
North-east	71.1	(63.1 – 78.0)	219	9.7	(6.1 – 15.1)	39	5.9	(3.2 – 10.7)	22	13.3	(8.7 – 19.9)	39	
Centre-east	67.9	(59.9 – 75.0)	199	8.9	(5.6 – 13.9)	36	9.8	(5.2 – 17.7)	21	13.3	(9.3 – 18.8)	48	
South	78.0	(73.5 – 81.9)	675	7.0	(5.1 – 9.3)	87	6.1	(3.9 – 9.4)	62	9.0	(6.4 – 12.4)	76	

CI= Confidence interval
